# An online mindfulness intervention for medical students in South Africa: A randomised controlled trial

**DOI:** 10.4102/sajpsychiatry.v28i0.1840

**Published:** 2022-05-27

**Authors:** Nicola Boyd, Debra G. Alexander

**Affiliations:** 1 Department of Psychiatry, Faculty of Medicine and Health Sciences, Stellenbosch University, Cape Town, South Africa

**Keywords:** online mindfulness-based interventions, medical students, well-being, perceived stress, self-compassion

## Abstract

**Background:**

Prior to the COVID-19 pandemic, an association was observed between medical students’ stress, possibly because of an intensive academic workload and clinical responsibilities, and mental ill health. The literature has shown the benefit of online mindfulness interventions for different mental health challenges. Unfortunately, there is a paucity of information on their benefit to medical students in South Africa.

**Aim:**

The aim of this study was to explore whether medical students attending an online mindfulness-based intervention (MBI) would show improved resilience and stress management compared with attendance at an online supportive counselling (SC) programme. Secondary to this was the viability of the intervention, for which an in-depth understanding of participants’ experiences was sought.

**Setting:**

The study setting was online through https://zoom.us/.

**Methods:**

Forty-five participants were randomly allocated between two 6-week, teacher-facilitated groups. A repeated measures analysis of variance (ANOVA) of outcome, well-being, perceived stress and self-compassion scores conducted at three time points, as well as thematic analysis of participant feedback, contributed to quantitative and qualitative data.

**Results:**

Participants in both the groups showed significant improvement over time in measures of well-being, perceived stress and subjective stress management. Participants in the mindfulness group showed a statistically significant treatment effect in mindfulness at programme completion. A decrease in self-compassion over time was observed in both the groups.

**Conclusion:**

The results of this study indicate that in this South African medical student cohort, an online MBI and a SC programme are both feasible and show potential for reducing stress, increasing stress management and increasing resilience. Further study in this area is recommended.

## Introduction

The COVID-19 pandemic severely impacted the global community and in particular healthcare systems. Medical education transitioned largely online to protect ill-equipped medical students from moral trauma,^1^ disease exposure and to stop the spread of the virus.^2^ Pre-pandemic, studies both internationally^3,4^ and locally,^5,6,7,8^ indicated an association between medical students’ increased stress and mental ill health. The pandemic further highlighted a need for specific knowledge curricula^9^ with associated resiliency interventions.^1^

Protection from the effects of stress is linked to high levels of resilience,^10^ which includes a positive adaptive capacity to adjust to challenging experiences^11^ and learned life skills^12^ in order to facilitate recovery and growth.^11,12,13,14^ Resilience-building programmes like mindfulness-based interventions (MBIs) for healthcare workers^14^ increase mutual support and reduce isolation, while within education, it increases well-being and stress resilience for students.^15,16^ The qualities of non-judging, curiosity and present-moment mindful awareness of an experience have the potential to change a person’s relationship to an experience.^17^

Internet-based interventions for increased mental health are cost-effective, accessible, convenient, effective and non-stigmatising.^18,19^ Aims are varied: to prevent,^18^ to treat a mental disorder^20^ and/or to promote well-being.^21^ Like in-person MBIs, Internet-based MBIs (IB-MBIs) have shown improved mental well-being,^22^ reduced anxiety, depression and stress.^19,20,23^ Formats, content, programme and session length vary widely depending on the target population.

For students, IB-MBIs are a popular method to introduce stress management,^21,24^ as they are familiar with accessing information online^25^; however, rates of attrition are high.^21,24,26^ Increased contact between participants and facilitators has been recommended to reduce attrition.^27^

Although research studies are limited, IB-MBIs have shown benefits for time constrained, medical students who may be located off-campus on clinical rotation^28,29,30^ or at universities without mindfulness programmes.^31^ Increased awareness and presence, self- and compassion for others, as well as improved emotional regulation and stress management, have been reported.^28^ Time constraints, disliked content and prioritising daily practice were described as barriers to practice.^28,30^

Prompted by a need for potential resilience development programmes for South African medical students, the study aimed to investigate the feasibility and benefits of a teacher-facilitated, on-campus, 6-week MBI comparative to supportive counselling (SC). This was halted by South Africa’s implemented lockdown but continued in a different format: online.

## Aims and objectives

This study aimed at exploring whether a 6-week MBI in comparison with SC can increase resilience to stress within a sample of MBChB students. The objectives of this study were to increase participant well-being, increase self-compassion, and reduce stress as an indication of increased resilience to stress. In order to assess participants’ engagement with the programme and their change in stress management within this context, an in-depth understanding of their experiences was sought by collating data regarding the recruitment, programme completion, regularity of home practice, perceived value, as well as the suitability of the online data collection method.

## Research methods and design

### Study design

A mixed-method randomised controlled design was used. Participants were assessed at three time points using four online, self-reported quantitative measures, as well as a qualitative, self-reported, feedback form following the course completion.

### Study setting

This intervention was conducted online via https://zoom.us/.

### Study population and sampling strategy

A convenience sampling strategy was used with the following inclusion criteria:

- year 2–6 MBChB students (approximately *N* = 1540) who were currently registered at a Medical and Health Sciences Faculty in the Western Cape at the beginning of Semester 2, 2020
- aged 18 years or over
- a willingness to attend five of 6 weekly online sessions
- a willingness to complete 10–15 min of daily home practice
- access to the Internet and a laptop.

As the mindfulness programme involves observing internal experiences for periods, an exclusion criterion included any person who was currently experiencing severe depression, anxiety or other severe mental illnesses (as self-reported in the pre-course assessment).

A recommendation by Torgerson and Torgerson^32^ informed the sample size for which a minimum of 32 participants would enable the observation of one standard deviation (s.d.) difference between two randomised groups with 80% power. Forty-five students were recruited to allow for attrition.

Students received a flyer via email and class group WhatsApp. Three 30-min, online (zoom) information sessions were hosted. Interested students contacted the researcher by email and received information detailing the possible effects, potential benefits and the right to self-withdraw. The communication included an informed consent letter, which was signed by potential participants and returned by email to the researcher. Following which, potential participants completed an online, self-assessed, pre-course assessment from an emailed link. Thereafter, individual, 20- to 30-min WhatsApp video or zoom feedback sessions were completed. Non-eligible students were encouraged to contact Campus Health or their Mental Health Practitioner. Thereafter, de-identified participants’ numbers were randomised at a ratio of 2:1 (intervention: control) by a person independent from the study with a random number generator (commentpicker.com/teamgenerator.php). Apart from an offer to SC participants of an MBI following completion of the study, no inducements to participate were made.

### Interventions

#### Structure

Extracurricular, structurally equivalent, concurrent, 60-min, online programmes were facilitated via zoom. Emailed post-session summaries included a link to a daily home practice for MBI participants and an offer to provide extra support. Participants excused themselves if they were not able to attend a session. The virtual classroom remained open for 5–10 min post-session for participants who wished to ask questions or to seek individual assistance.

#### Mindfulness-based intervention content

The 6-week MBI was adapted from Williams and Penman’s 8-week self-help programme.^33^ Theme-based sessions consisted of practices, activities and enquiries. Early sessions focused on attention to internal and external experiences without judgement.^33^

Shortened practices included body and breath, mindful movement and the body scan.^33^ Practices provided opportunities to recognise mind habits, such as autopilot, mind wandering and dreaming. Mini meditations introduced the Three Step Breathing Space (3SBS) separately before including it in its entirety in week 3.^33,34^ The remaining sessions focused on learning to view thoughts as mental events, with practices like sounds and thoughts and exploring difficulty.^33^ Attitudes of kindness and curiosity for the self, personal experiences and other people were encouraged throughout the programme but were encapsulated within a Loving Kindness meditation in session 5.^33^

#### Supportive counselling content

The 6-week SC psychoeducational programme consisted of common, evidence-based stress reduction techniques used in psychotherapy. An introduction to stress included the use of a stress wheel and awareness triangle exercises.^35,36,37^ Thereafter, sessions focused on the role of emotions in heightening stressful experiences in conjunction with the awareness triangle to explore such experiences.^38,39,40,41,42^ Progressive muscle relaxation, journaling, a communication genogram, and identification of goals and values are examples of skills, which were shared as additional stress management skills.^43,44,45,46,47,48,49,50^ This took place within an encouraging, supportive environment, where participants reflected on the previous, present and future challenges in conjunction with psychoeducation and skills’ practice.

### Data collection

Measures were completed online at pre-, post-course and 8-week follow-up (8WFU) on password protected, Microsoft Office Forms.com. This included (1) sample demographic characteristics (age, gender and year of study), (2) Clinical Outcomes in Routine Evaluation – Outcome Measure (CORE-OM),^51^ (3) Warwick-Edinburgh Mental Well-being Scale (WEMWBS),^52^ (4) Perceived Stress Scale (PSS),^53^ (5) Self-Compassion Scale – short form (SCS-sf)^54^ and (6) feedback form (post-course). The CORE-OM was used as a pre-course assessment. Participants’ well-being was tested mid-programme with the WEMWBS. If participants’ WEMWBS score decreased by three to eight points (indicating a reduction in mental well-being), contact was made for an appropriate referral. As elsewhere,^16^ South African studies with students have utilised both the CORE-OM and WEMWBS.^55,56^

#### Clinical Outcomes in Routine Evaluation – Outcome Measure

Symptoms of distress were measured with the user-friendly, standardised, CORE-OM.^51^ The 34-item scale includes positive characteristics and symptoms of mental ill-health in areas of well-being, problems or symptoms, functioning and risk. Examples include ‘I have felt optimistic about my future’, ‘I have felt able to cope when things go wrong’ and ‘I have felt criticised by other people’.^51^ A mean score is assessed for total (range 0–40) and individual domain scores. Internal consistency is high (α = 0 > 0.75, < 0.95), test–retest reliability is good for all domains, except for risk (0.87–0.91, risk = 0.64). Sensitivity to change following an intervention is described as ‘good’.^57^

#### Warwick–Edinburgh Mental Well-being Scale

Participants’ well-being during the past 2 weeks was measured with the self-assessed, 14-item, mental well-being monitoring instrument, WEMWBS.^52^ Positive statements about thoughts and feelings include ‘I’ve been dealing with problems well’ and ‘I’ve been feeling confident’, which are answered on a five-point Likert scale. Scores range from 14 (minimum) to 70 (highest) with item responses summed. Strengths include its simplicity, item clarity, sensitivity to change, good internal consistency (α = 0.89) and high test–retest reliability (0.83 at 1 week).^58^ It corresponds closely with other mental well-being scales.

#### Perceived Stress Scale

The clearly worded, widely used PSS was used to assess participants’ self-reported, perceived current level of stress during the last month.^53^ A five-point Likert scale ranges from ‘0’ (never) to ‘4’ (very often), with higher scores reflecting more perceived stress. Of the 14 items, seven are positively worded and are reverse scored. An example of this include ‘In the last month, how often have you felt that things were going your way’. All items are summed for a total score with a minimum of 0 (no stress) and a maximum of 40 (highest stress level). It has adequate internal consistency (α = 0.84, 0.85 and 0.86 for three samples).^53^

#### Self-Compassion Scale – short form

The SCS-sf’s 12 statements reflect Neff’s^59^ proposed definition of self-compassion, including self-kindness, common humanity and mindfulness, as well the reverse: self-judgement, isolation and over-identification.^54^ Statements like ‘When I feel inadequate in some way, I try to remind myself that feelings of inadequacy are shared by most people’, and ‘I’m disapproving and judgmental about my own flaws and inadequacies’ are rated from 1 to 5. The negative subscale items are reverse scored before calculating the mean total score.^54^ Useful with time-constrained participants, the SCS-sf has adequate internal consistency (α = 0.86), correlates closely the Self-Compassion Scale (long form) (α = 0.97) and is reliable when assessing the overall self-compassion scores in time-constrained settings.^54^

#### Feedback form

A post-course feedback form recorded participants’ perspectives of their experience, including their reasons for having volunteered and how the course was helpful to them. Four scales, two related to self-reported adherence to daily home practice (per week and per day of formal and informal practices) and two Likert scales (1 [poor] – 5 [excellent]) for programme and resource ratings, were included. The data were analysed using SPSS. In order to monitor attendance, a register was kept for each session.

### Data analysis

The first author received the raw data, which was stored on a password-protected computer. De-identified quantitative and qualitative data was sent to a faculty Biostatistician, and an independent qualitative researcher respectively, for analysis. Cycles 1 and 2 were analysed simultaneously. A repeated measures analysis of variance (ANOVA) with two- and three-time point analyses were conducted using SPSS. Treatment- and time effects for the groups were assessed. A Wilk’s lambda test statistic was reported for all, except Risk. A Mann–Whitney *U* test statistic was reported for change in scores between groups and the Wilcoxon signed ranks statistic for time effect, for risk. For qualitative analysis, codes and themes were determined independently of the first author.

### Ethical considerations

Ethical approval was obtained (reference No. S19/05/104, 22 August 2019). Following lockdown, ethical permission was again sought and received for online participation and inclusion of Years 2 and 6 MBChB students (reference No. S19/05/104, 26 June 2020). Permission was received to work with the students from the relevant authorities.

## Results

A total of 45 MBChB students consented to participate in the study. Five participants self-withdrew: three before randomisation (not eligible) and two during the programme because of ill health and Internet connectivity constraints. Thirty-eight participants (age range 19–30 years) completed the programmes and all measures. Women participants (71.1%) outnumbered men. Year 4 participants were in the majority (39.5%), followed by Year 2 (21.1%), Year 5 (18.4%) and Year 6 (13.2%), with Year 3 the least represented (7.9%).

### Quantitative results

The results for time and treatment effects are recorded in Table 1. Statistical significance for all tests was set at *p* < 0.05. The CORE-OM (Total), WEMWBS, and PSS results indicated significant improvement for both the groups over time (*p* < 0.01) at post-course and 8WFU. This contrasted with both groups’ SCS-sf (Total) result, which reflected a significant decrease over time, post-course and 8WFU (*p* = 0.004; *p* = 0.016). The mindfulness subscale of the SCS-sf reflected as a treatment effect between the groups (*p* = 0.045) at post-course only.

**TABLE 1:** Time- and treatment effects for Clinical Outcomes in Routine Evaluation – Outcome Measure, Warwick–Edinburgh Mental Well-being Scale, Perceived Stress Scale and Self-Compassion Scale – short form (*n* = 38).

Effect	Treatment				Time			
	Post-course analysis		8-week follow-up		Post-course analysis		8-week follow-up	
	Statistic†	*P*	Statistic†	*P*	Statistic†	*P*	Statistic†	*P*
**CORE-OM (Total)**	1.000	0.927	0.992	0.865	0.585	< 0.001*	0.550	< 0.001*
Total-risk	1.000	0.915	0.993	0.883	0.606	< 0.001*	0.577	< 0.001*
Subscales	-	-	-	-	-	-	-	-
Well-being	0.941	0.137	0.912	0.201	0.866	0.022*	0.859	0.070
Problems	0.993	0.624	0.995	0.910	0.460	< 0.001*	0.448	< 0.001*
Functioning	0.999	0.855	0.954	0.439	0.998	0.784	0.958	0.475
Risk	136.500†	0.263†	126.000†	0.212†	10.000‡	0.002‡*	11.000‡	< 0.001‡*
**WEMWBS**	0.997	0.728	0.989	0.825	0.525	< 0.001*	0.522	< 0.001*
**PSS**	0.995	0.668	0.987	0.789	0.554	< 0.001*	0.450	< 0.001*
**SCS-SF (Total)**	0.984	0.447	0.947	0.384	0.789	0.004*	0.789	< 0.016*
Subscales	-	-	-	-	-	-	-	-
Self-kindness	0.939	0.135	0.931	0.286	0.877	0.031*	0.561	< 0.001*
Self-judgement	0.925	0.097	0.925	0.257	0.896	0.049*	0.819	0.030*
Common	0.965	0.262	0.965	0.535	0.911	0.068	0.802	0.021*
Humanity	-	-	-	-	-	-	-	-
Isolation	0.995	0.679	0.907	0.183	0.763	0.002*	0.693	0.002*
Mindfulness	0.893	0.045*	0.886	0.119	0.923	0.091	0.892	0.135
Over-identified	0.964	0.252	0.963	0.517	0.601	< 0.001*	0.518	< 0.001*

CORE-OM, Clinical Outcomes in Routine Evaluation – Outcomes Measure; WEMWBS, Warwick–Edinburgh Mental Well-being Scale; PSS, Perceived Stress Scale; SCS-sf, Self-Compassion Scale-short form.

* , Statistical significance for all tests was set at *p* < 0.05.

† , Wilk’s lambda test statistic is reported for all except Risk. For Risk, the Mann–Whitney *U* test statistic is reported for change in scores between groups.

‡ , Wilk’s lambda test statistic is reported for all, except risk. For risk, the Wilcoxon signed ranks test statistic is reported.

### Qualitative results

Participants’ responses from the feedback form were collated and sorted into themes and subthemes. For each group, four main themes were identified. Table 2 and Table 3 show the themes, subthemes and corresponding participants’ statements. The results of the MBI participants will be reported first.

**TABLE 2:** Themes, subthemes and statements of the mindfulness group.

Theme	Subtheme	Statement
1. Change in the initial stress reaction	1.1. Pause and breathe 1.2. Step back from destructive thought processes 1.3. Acknowledge that I am not my thoughts: regain control	1.1.1. ‘I have slowed down & allowed the “pause” practice to help me when I start to become overwhelmed… to manage moments rather than everything at once.’ 1.2.1. ‘(I) use the approach of kind curiosity to explore thoughts, feelings & body sensations I experience.’ 1.2.2. ‘I now do a breathing exercise to calm myself down, realise that if I messed up, that is ok.’ 1.3.1. ‘I practiced the 3SBS whenever I felt anxious or overwhelmed & stressed while studying. This practice did not reduce the occurrence of these feelings but definitely enabled (sic) me to be cognisant & manage it in a more effective & quicker way.’
2. Positive change in relationships	2.1. Kinder to self 2.2. Improved interaction with others	2.1.1. ‘The session in which we expressed words of affirmation & love to ourselves & other beings, truly moved me. A lot of my stress is related to my self-critical nature & so it’s been very interesting & good for me to try & be more gentle & loving towards myself.’ 2.2.1. ‘Manage conflict better… disagreements can be resolved more-calm (sic) & effectively.’ 2.2.3. ‘My improved mood also led to me wanting to socialise more … (I) felt more capable of supporting others when they were struggling with something.’
3. Feeling of a sense of community through course interactions	3.1. Normalisation of feelings and experiences 3.2. Supportive facilitation	3.1.1. ‘(I) found listening to others’ experiences & perspectives to be helpful for my own growth… experience challenges together & know you are not alone.’ 3.1.2. ‘Every week there was one or two people that I could relate to EXACTLY what they were saying.’ 3.2.1. ‘(The Facilitator was) non-judgmental, sets good boundaries & is punctual & respectful of time constraints. These qualities made it feel like a safe & trusted environment.’ 3.2.2. ‘Friendliness & non-hostility… there was no pressure on us for once, so it didn’t feel like a chore. I always looked forward to the sessions.’
4. Time as a barrier to practice	4.1. Having time and space 4.2. Making time and building a habit 4.3. Ease of incorporating informal practices	4.1.1. ‘A lot of interruptions at home.’ 4.1.2. ‘Struggled to find a quiet place.’ 4.1.3. ‘But over time, adjusted my time of when to do some of the practices in such a way that I get that peace & quiet.’ 4.1.4. ‘Being on call & working strange hours.’ 4.2.1. ‘The weekends were very difficult since I usually did the formal ones early in the morning during the week before hospital.’ 4.2.2. ‘However, once I was in the routine of things & the practices became more familiar, participation became more enjoyable.’ 4.2.3. ‘Confronting some very difficult personal issues made formal scheduling quite difficult.’ 4.3.1. ‘Helpful…because it allowed me to include & practice mindfulness in my daily life.’ 4.3.2. ‘It does not take up too much time & even just a little bit of practice makes the world of difference.’ 4.3.3. ‘I’d make sure to practice these whilst walking to hospital, centering (sp.) myself before presenting to a doctor etc. This was easier.’ 4.3.4. ‘Implemented more often & in many different contexts & spaces.’

**TABLE 3:** Themes, subthemes and supportive counselling participants’ statements.

Theme	Subtheme	Statements
1. Awareness of emotions and reactions	1.1. Awareness triangle was most helpful 1.2. Awareness improved 1.3. Use of skills	1.1.1. ‘(It) helped me deconstruct my anxiety into manageable pieces. This calmed me down and enabled me to come out with a plan to reduce my anxiety.’ 1.2.1. ‘I know now that what works for me is to have a routine of a more balanced activities to keep from the overwhelming feeling and excessive procrastination.’ 1.2.2. ‘I’m just a lot more conscious of how my emotions can affect not just me but the people around me.’ 1.2.3. ‘There is no use projecting my frustrations and stress onto the people around me – yes, they can be there to help when appropriate, but irritable mood and negativity tends to push people away.’ 1.3.1. ‘The other spokes of the wheel and how they contribute to my mental health and balance… (resulted in being) more-gentle (sic)/ kind/caring towards myself and more understanding of my limitations.’ 1.3.2. ‘I am aware of negative thoughts (,) and I try to write my thoughts down and also the solutions so that my anxiety is not so bad anymore because I have realised that most solutions work. So, I don’t panic anymore thinking that everything is falling apart.’
2. Change in approach to dealing with emotions: do not be consumed	2.1. Analyse the situation and find solutions	2.1.1. ‘I analyse the situation by figuring out if it is something in or out of my control… if it is something in my control, I come up with solutions to alleviate my stress.’ 2.1.2. ‘I have been submitting my assignments a lot earlier… I don’t have deadlines looming over my head.’
3. Increase in consciousness in relationships with others		3.1.1. ‘I feel more comfortable telling those close to me when I am stress(ed) or anxious & that has been relieving.’ 3.1.2. ‘I have helped a few people to overcome acute stress.’
4. Barriers to engaging with tools	4.1. Emotions 4.2. Time	4.1.1. ‘I avoided sitting down to work on them because I struggled to feel the emotions and prefer avoiding them. It meant that I would then prefer to just think about them then formally engage with them.’ 4.1.2. ‘Relationship difficulties sometimes… even a low moods day.’ 4.2.1. ‘Sometimes you’ll find there isn’t time or just too tired from the day & discouraged to put in the extra effort.’

The themes of the MBI group’s participants reflected positive changes in their initial stress reaction (theme 1), their relationships (theme 2) and a feeling of being part of a community because they had participated in the course (theme 3). The group reported experiencing several barriers to the development of a practice (theme 4).

#### Themes, subthemes and statements of the mindfulness group

**Theme 1: Change in the initial stress reaction:** A positive change in participants’ initial stress reaction was associated with the use of different practices like The Pause (1.1.1), or 3SBS (1.3.1), and/or the adoption of a self-compassionate attitude (1.2.1). Nine participants reported feelings of being calmer and grounded, while 14 participants reported use of the practices, which contributed to their changed response. Participants’ increased self-awareness made it possible to de-centre from unhelpful mind habits (1.2), which contributed to increased personal agency (1.3.1). One of the participants reported that:

‘Knowing I have the tools to help me during stressful situations has helped me tremendously. I would be afraid of stress as I knew I did not know how to deal with it well.’ (P38)

**Theme 2: Positive change in relationships:** Mindfulness-based intervention participants perceived that the mindfulness training had positively affected their intra- and interpersonal relationships.

Fourteen participants observed that they had noticed that they were experiencing less self-judgment, self-blame, self-anger and self-disappointment. Instead, increased consciousness, self-care and self-compassion (2.1.1.) were reported. An example of this includes:

‘I also make time to give myself small acts of kindness… and I find this really enjoyable.’ (P20)

Some participants attributed their increased self-kindness and associated improved mood to the positive changes in interpersonal relationships. Such changes involved a willingness to listen, to offer support (2.2.3), to be more understanding, as well as improved conflict management (2.2.1.). One of the participants reported that:

‘I can notice changes in my behaviour, like I don’t easily get irritated with people around me.’ (P34)

**Theme 3: Feeling of a sense of community through course interactions:** While the programme was offered during the COVID-19 lockdown, 11 participants in the MBI group reported feeling a sense of community as they met weekly and listened to each other’s experiences (3.1.1; 3.1.2.). One of the participants described this as being on the same journey together, while another found that:

‘Knowledge is helpful… I realise it is normal and just my brain’s way of dealing with things in my life.’ (P12)

Twelve participants found that the supportive way the programme was facilitated had contributed to their appreciation of the mindfulness training. The online context was noted as safe, comfortable (3.2.1), unpressured (3.2.2), as well as kind and encouraging. The presence of a facilitator was helpful as noted by this participant:

‘I appreciated real-time feedback and clarification of any concerns or questions.’ (P20)

**Theme 4: Time as a barrier to practice:** The medical students reported that time was the main barrier to daily formal practice but less so for the informal practices. It was found that for 17 participants, the latter could be included in daily life with more ease (4.3.1–4.3.4). Twenty-two participants reported having experienced difficulties with time management, a physical space in which to practice (4.1.1–4.1.4), as well as emotional challenges which arose during meditation (4.2.3). Regular practice was associated with flexibility in relation to time and space (4.2.2), as well as the choice of practice as can be observed in the following two participants’ comments:

‘I thought that since I did the formal practices, it would be ok if I did not always do the informal practices.’ (P20)

This contrasts with the comment:

‘Because of my busy schedule I found it difficult to set aside a specific time for (formal) mindfulness (practice). Instead, I incorporated some of the shorter practices (informal practices) into my daily activities to make sure that I practice.’ (P27)

As presented in Table 3, the themes, subthemes and statements of the SC participants are discussed. For participants, the programme contributed to an increased awareness of emotions (theme 1), changes in the way that emotions were managed (theme 2) and an increased consciousness in relationships with others (theme 3). Like the MBI group participants, the SC group reported that there were barriers to their regular use of the tools. Four participants reported that their management of stress was unchanged at the programme’s conclusion.

#### Themes, subthemes and supportive counselling participants’ statements

**Theme 1: Awareness of emotions and reactions:** One of the themes identified from 11 of the SC participants’ responses was that there had been an improvement in the awareness of their emotions and associated reactions (1.2.). Increased self-awareness had contributed to increased self-agency and self-kindness (1.1.1.–1.3.2.). The technique, which nine participants reported was associated with this change, was the awareness triangle (1.1.1.). As one of the participants reported:

‘The triangle, because it will allow me to sit down and actually sort out my feelings, instead of one overwhelming emotion and therefore work out situations to the best of my ability.’ (P36)

**Theme 2: Change in approach to dealing with emotions: Do not be consumed:** The SC participants reported feeling calmer, relieved and more effective in managing their emotions. This increased self-compassion and self-agency were associated with analysing a situation and identifying solutions (2.1.1 and 2.1.2.) and/or flexibility in tool selection and use. This is visible in the following responses:

‘I have been implementing some of the tools taught to us and they have generally made me calmer and allowed me to take a more balanced and understanding approach to my emotions and how to manage them.’ (P29)

In addition:

‘I have a better approach to handling certain situations such as dealing with anger. I have a better approach to dealing with this emotion now instead of bottling it up and leaving it to fester. I have adopted the expressive writing tool as a form of emotional relief.’ (P5)

**Theme 3: Increase in consciousness in relationships with others:** Theme 3 observed an increased interpersonal interaction between SC participants and their family and friends. This was characterised by some reporting that they experienced comfort from others when self-disclosing about their challenging experiences and/or emotions (3.1.1) and others reporting that they had helped others with their stress management (3.1.2). Another reported an intention to:

‘Try to make it a habit to improve interpersonal relationships.’ (P8)

**Theme 4: Barriers to engaging with tools:** The barriers to more frequent use of the techniques which SC participants reported they had experienced during the programme were the presence of challenging emotions, time constraints, relationship difficulties, family responsibilities, fatigue and daily life interruptions. Seven participants reported time constraints (4.2.1.) as their most significant barrier to technique usage, followed by emotional challenges (4.1.1.–4.1.2.). This resulted in an attitude of avoidance as was noted by one of the participants that:

‘I’m not ready for some of these practices eg journaling but I’m slowly working my way towards being more open to trying them out.’ (P39)

### Feasibility

Participants’ primary self-reported motivation for programme participation was to address the perceived lack of stress management skills. A desire to learn emotion-regulation strategies and a curiosity about mindfulness were additional motivators. Attendance was good with participants, on average, attending four of six sessions. After completing the MBI online, participants retrospectively reported having practised, on average, 3.6 formal practices per week, and 2.5 informal practices per day. This was higher than the average of 2.3 skills per week, practised by SC participants. Both the programmes were rated above average, with MBI resources rated more highly (*M* = 5, s.d.: 0) than the SC programmes (*M* = 4, s.d.: 1).

## Discussion

This randomised controlled pilot study conducted at a South African medical school investigated the benefit of an online teacher-facilitated MBI to increase medical students’ resilience to stress.

The results revealed significant improvement in both groups’ well-being (*p* < 0.001), and how they perceived and managed stress (*p* < 0.001), supporting findings in other studies.^21,27,60,61,62^ A statistically significant difference between participants in mindfulness (*p* = 0.045) reflected this changed stress management post-course.

Qualitative data revealed that MBI participants had begun to learn to decentre from and to adopt a metacognitive awareness in response to stress. These are mechanisms whereby the practice of mindfulness can affect change.^60,63^ This occurred through experiential practices (theme 1), real-time facilitator feedback (subtheme 3.2) and increased social support (theme 3) from group-oriented, extra-curricular activities with increased self-agency, confidence in stress management and well-being^21,22,61,64^

The encouraging quantitative and qualitative findings contrast with a statistically significant decrease in participants’ self-compassion. The literature reports varied results in self-compassion following MBI interventions: most frequently, increased self-compassion for others,^28,65,66^ less frequently, others not,^67,68^ possibly because self-compassion practices affect each person differently.^69^ The possible reasons for this result may be the medical students’ participation increased the awareness of lack of self-care and self-compassion.

## Study limitations and strengths

Limitations of the study include the exclusion of participants because of lack of data or connectivity issues, under-representation of Year 3 participants, over-representation of women participants, and small sample size limiting the generalisability of results, and secondly, the role of the first author as both the researcher and the facilitator. Although the first author’s simultaneous role may have provided programme consistency, her training, language and implicit stance as a mindfulness-based registered counsellor may have unintentionally affected the programme results.

In order to support the study’s credibility, mixed-method triangulation and assistance with independent data analysis were utilised.

The study’s strengths are the flexibility with which the programmes transitioned online, low attrition, good online response rates and participants’ engagement with the programme and practices that contrasted with other studies.^21,24,26,28^ The mixed-method design countered participants’ test effect and thematic analysis contributed to quantitative results providing additional information about participants’ self-compassion, interpersonal relationships and increased mindfulness in the MBI group. The inclusion of an active control indicated that mindfulness training can increase resilience, as well as a psychoeducational SC programme. This contrasts with studies that had an inactive or no control.^16,28^

## Recommendations

As both groups’ results indicated increased resilience, a programme of wellbeing enhancement for medical students is recommended. This could be provided for within the curriculum, as an extramural or through Campus Health services increasing access to psychological support.

More specifically, in terms of the MBI offering, it would be helpful to include booster sessions post-programme to maintain benefits. Implementation of a safe MBI environment necessitates facilitation by a suitably qualified mindfulness teacher with appropriate experience as suggested elsewhere.^70,71,72^

Research, which is extended to other medical education facilities, is recommended as well as the benefit of mindfulness training to address medical student burnout, fatigue and suicidality; long-term mindfulness practice benefits post-qualification and change in self-compassion at longer follow-up.

## Conclusion

This online MBI pilot study showed promising results. There is a need for innovative, feasible and accessible stress management skills and resiliency interventions programmes for medical students in South Africa. Further studies, which build on this work, are recommended.
